# Incremental Ramp Load Protocol to Assess Inspiratory Muscle Endurance in Healthy Individuals: Comparison with Incremental Step Loading Protocol

**DOI:** 10.21470/1678-9741-2023-0231

**Published:** 2024-02-26

**Authors:** Guilherme de Souza Areias, Alexandre Fenley, Luan Rodrigues Santiago, Alessandra Choqueta de Toledo Arruda, Rodrigo Boemo Jaenisch, Solange Guizilini, Michel Silva Reis

**Affiliations:** 1 Grupo de Pesquisa em Avaliação e Reabílitação Cardiorrespíratória (GECARE)/Faculdade de Fisioterapia, Programa de Pós-Graduação em Educação Física/Escola de Educação Física e Desportos, Uníversídade Federal do Río de Janeíro, Río de Janeiro, Río de Janeiro, Brazil; 2 Grupo de Pesquisa em Avaliação e Reabilitação Cardiorrespíratória (GECARE)/Faculdade de Físíoterapía/ Programa de Pós-Graduação em Medícina-Cardíología/Instítuto do Coração Edson Saad, Uníversidade Federal do Rio de Janeíro, Rio de Janeíro, Rio de Janeíro, Brazíl; 3 Departamento de Físioterapía e Reabílítação, Programa de Pós-Graduação em Ciências do Movímento e Reabílítação, Uníversídade Federal de Santa María, Santa Maria, Rio Grande do Sul, Brazíl; 4 Programa de Pós-Graduação em Cardíologia, Uníversídade Federal de São Paulo, São Paulo, São Paulo, Brazíl

**Keywords:** Exercise Test, Respiratory Muscles, Physical Endurance

## Abstract

**Introduction:**

Protocols for obtaíníng the maxímum threshold pressure have been applied wíth límited precision to evaluate ínspiratory muscle endurance. In thís sense, new protocols are needed to allow more relíable measurements. The purpose of the present study was to compare a new incremental ramp load protocol for the evaluation of ínspíratory muscle endurance wíth the most used protocol in healthy indíviduals.

**Methods:**

This was a prospective cross-sectional study carried out ín a síngle center. Nínety-two healthy indíviduals (43 men [22 ± 3 years] and 49 women [22 ± 3 years]) were randomly allocated to perform: (i) íncremental ramp load protocol and (íí) íncremental step loadíng protocol. The sustained pressure threshold (or maximum threshold pressure), maximum threshold pressure/dynamic strength índex ratío, time untíl task faílure, as well as dífference between the mean heart rate of the last five mínutes of baselíne and the peak heart rate of the last 30 seconds of each protocol were measured.

**Results:**

Incremental ramp load protocol wíth small íncreases in the load and starting from mínímum values of strength index was able to evaluate the inspiratory muscle endurance through the maxímum threshold pressure of healthy indívíduals.

**Conclusion:**

The present study suggests that the íncremental ramp load protocol is able to measure maximum threshold pressure in a more thorough way, wíth less progression and greater accuracy in the load stratification compared to the límited incremental step loading protocol and with a safe and expected cardiovascular response in healthy individuals.

## INTRODUCTION

The inspiratory muscle training (IMT) has been applied to improve the capacity of inspiratory muscles to generate maximum force (strength) and to maintain a specific muscular task over time (endurance)^[1]^. The inspiratory muscle resistance training protocols have been shown to be effective in patients in the cardiovascular postoperative period^[1,2]^ and in those with chronic heart failure (CHF)^[1,2]^, chronic obstructive pulmonary disease^[3,4]^, asthma^[5]^, and chronic kidney disease^[6]^. The measurement of maximum inspiratory pressure (MIP) had been commonly used as a simple assessment of global inspiratory muscle strength (IMS), however, its physiological benefits to prescribe IMT have been questioned for healthy individuals and athletes who do not present inspiratory muscle weakness. Hill et al.^[7]^ questioned the relevance of MIP for not mimicking an effort commonly obtained in the daily life activities of individuals with or without some respiratory disorder. Therefore, considering that the mechanism involved in the optimization of the IMS of individuals without muscle weakness is related to reduction of the oxygen supply and to the competition for the peripheral blood flow at the peak of the exercise, it is possible to question the relevance of some training protocols for IMS of these individuals. The prescription of IMT should be aimed at improving inspiratory muscle endurance (IME) in order to improve inspiratory muscle tolerance to strenuous exercise and decrease inspiratory muscle metaboreflex^[8]^.

Studies have shown a significant increase of sustained pressure threshold during an incremental test (maximum threshold pressure [PthMax]) using MIP as the basis of IMT^[9,10]^. However, IME evaluation protocols are used only for post-intervention analysis^[7,9,11]^. This allows to envisage the benefits of IMT protocol based on the previous IME evaluation and by using the PthMax as an IMT prescription parameter and not as a variable to just control the training effects. Given the potential of the PthMax, a good protocol to obtain loads is necessary. The protocol for evaluating IME through PthMax is based on a progressive increase of the load in percentage platforms and has been shown to be an important and widely used tool^[6,10,12,13]^. However, the increase of loads on percentage platforms of stratification occurs every 10%, the threshold of failure during the test does not reflect, accurately, the exact values with which the individual would not support a certain ventilatory overload. Another factor is the threshold of the percentage of the test in which the individual fails. Since PthMax is only established when the subject performs more than half the time/incursions in the corresponding percentage platform, this implies the possibility of underestimating the IME of these individuals. A high initial load, around 50-60% of MIP, is another factor limiting the test for individuals with low endurance capacity.

PthMax is therefore a measure with enormous diagnostic and exercise prescription potential and is being underestimated, mainly due to a limited stratification protocol, which makes the test inappropriate for individuals with low IME. In this context, the objective of the present study was to compare a new tool for the evaluation of IME, with small load increases and starting at lower values of the dynamic strength index (S-Index), with the most used protocol within the method of pressure threshold loading.

## METHODS

### Study Design and Participants

This prospective cross-sectional study was conducted at laboratory of Grupo de Pesquisa em Avaliação e Reabilitação Cardiorrespiratória (GECARE), Faculdade de Fisioterapia, Universidade Federal do Rio de Janeiro. Participants were consecutively recruited between February 2018 and June 2020. This study was guided by the Strengthening the Reporting of Observational Studies in Epidemiology (or STROBE) statement^[14]^.

One hundred and one healthy individuals according to clinical evaluation of both sexes, aged between 18 and 40 years, were recruited from a university population. The volunteers were submitted to a detailed evaluation, in which the personal data, anthropometrics, and vital signs were collected. The anamnesis and physical examination were performed in order to investigate the history of previous diseases, as well as lifestyle and food habits. The individuals were stratified into very active, active, irregularly active, and inactive by the short form of the International Physical Activity Questionnaire (IPAQ)^[15]^. Individuals who were unable to perform the IMS protocol, participants with a history of smoking or illicit drugs consumption, and those with cardiovascular (such as systemic arterial hypertension, CHF, electrical conduction disorder, among others), respiratory (obstructive and restrictive diseases), muscular (myopathy), neurological, metabolic (diabetes mellitus) and immunological diseases were excluded. This sample size was justified by a priori power analysis in G*power using a target effect size of f = 0.25, alpha of 0.05, and power of 0.80, which determined that 36 subjects were required for participation; the additional recruitment accounted for the possibility of dropouts. This project was approved by the Ethical Committee of Hospital Clementino Fraga Filho, Universidade Federal do Rio de Janeiro (CAAE 43656115.8.0000.5257). All volunteers signed an informed consent form to participate in this research.

### Experimental Protocols

A prospective cross-sectional, blind, and randomized study was undertaken to compare an incremental ramp load protocol (IRLP) and an incremental step loading protocol (ISLP) (control condition). The individuals were submitted to the S-Index measurement by using an inspiratory linear charge resistor (Power Breathe, IMT Technologies Ltd, Birmingham, United Kingdom). After S-index measurement, individuals were randomly allocated to the incremental load protocols: (i) ISLP (control condition) and (ii) IRLP with a minimum recovery time of 15 minutes in between. For the comparison of the protocols, three variables were analyzed: (i) PthMax, characterized as the peak load reached during the incremental tests — it reflects a physical measure (cmH₂O) representative of its maximum IME capacity —; (ii) PthMax/S-Index ratio, characterized as the ratio between the IME and the pressure peak in isolated maneuver — this relationship is able to show if the individuals have a very low or low IME, because it normalizes the peak resistance with the data of the peak of pressure obtained, providing data in percentage —; (iii) total time (s), duration time of the individual in each protocol until task failure.

The same linear inspiratory resistor (Power Breathe, IMT Technologies Ltd., Birmingham, United Kingdom) was used to perform the protocols, and individuals received the same instructions from the S-Index evaluation for optimal test performance. Blood pressure was measured at the beginning and end of each incremental protocol.

### Dynamic Strength Index Measurement

The S-Index was determined after maximal inspiratory effort, from the residual volume to the total lung capacity, against a mouthpiece duly coupled to the volunteer. The S-Index values were those observed at the peak pressure generated by the pressure × time plot observed through BreatheLink Software 1.1. The S-Index was measured with a 30-second interval between maneuvers, being considered the highest value found in three reproducible maneuvers (difference < 10%)^[16]^. A short period of training was performed until the individual was familiar with the functioning of the device^[17,18]^. Individuals were instructed to use a nasal clip and to not perform compensatory head and trunk movements during the maneuver. We also performed a previous correlation and concordance analyze between serial S-Index and MIP measurements with a sample of 45 individuals with a strong and significant correlation (*r* = 0.74 and *P<*0.0001) and good agreement^[16]^.

### Incremental Step Loading Protocol

**Fig. 1:**
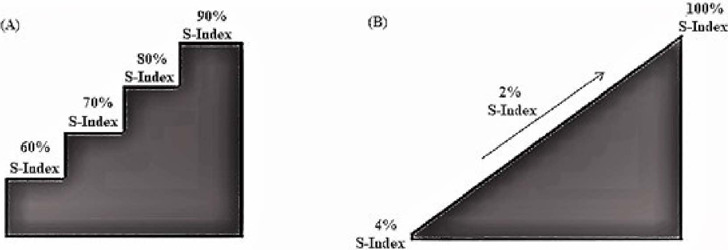
Illustration of the protocols for obtaining the sustained pressure threshold during an incremental test (maximum threshold pressure): (A) incremental step loading protocol; (B) incremental ramp load protocol. Values are expressed as a percentage of the dynamic strength index (S-Index).

The initial load was 60% of the S-Index, with 10% added every minute without interruptions between overloads. The maximum possible load to be reached was 90% of the S-Index or until interruption by failure, observed by the non-opening of the inspiratory valve by the individuals (Figure 1). The respiratory rate was controlled by verbal command^[19]^ of the same researcher during the test, being induced to 15 cycles per minute (inspiration:expiration ratio = 1:3). It was considered as the peak load, that the individual can perform at least seven full raids or 30 seconds on that percentage platform. Since the test aims at reaching failure, characterized by the inability to overcome the burden imposed during three consecutive inspirations^[20]^, there was no pause period between each percentage platform.

### Incremental Ramp Load Protocol

The initial load of this protocol is based on the lowest value provided by the device (3 cmH_2_O) and, in the sequence, incremental increases of 2% (less load progression according to the technical limitation of the resistive load S-Index at each ventilatory incursion until reaching 100% of the S-Index or failure interruption, characterized by the inability of the volunteer to overcome the load imposed during three consecutive inspirations) (Figure 1). The respiratory rate was controlled by the verbal command of the researcher during the test, being induced to 15 cycles per minute (inspiration:expiration ratio = 1:3).

### Cardiovascular Stress

Sixteen randomized individuals were selected to infer cardiovascular stress from each IME assessment protocol. Heart rate (HR) was collected, beat-to-beat, through the Polar® V800 cardio frequency meter^[21]^ during rest (10 minutes) and during ISLP and IRLP protocols. The difference between the mean HR of the last five minutes of baseline and the peak HR of the last 30 seconds of each protocol was considered for analysis. Additionally, systolic and diastolic blood pressures (SBP and DBP, respectively) were assessed before and after ISLP and IRLP protocols.

### Data Analysis

For statistical analysis, Sigmaplot Software 12.0 was used, and the volunteers were divided by sex. Initially, the Shapiro-Wilk and Levene tests were applied to evaluate normality and homogeneity, respectively. The PthMax/S-Index, PthMax, total time and HR Delta data were analyzed using the paired *t*-test. The Pearson correlation was then applied to evaluate the association between the protocols. The Bland-Altman analysis with a 95% confidence interval was adopted to evaluate the agreement of the PthMax between the protocols. Data were presented as mean and standard deviation with an established level of significance of *P<*0.05.

## RESULTS

**Fig. 2:**
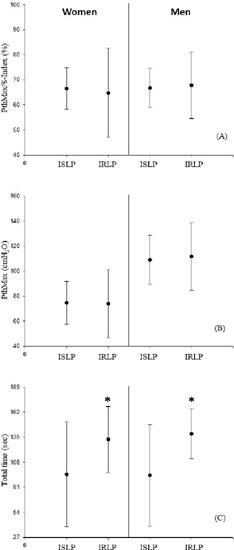
Comparison of incremental step loading protocol (ISLP) and incremental ramp load protocol (IRLP) by sex. (A) Sustained pressure threshold ratio during an incremental test (maximum threshold pressure [PthMax])/dynamic strength index (S-Index); (B) PthMax; (C) total time. *t-test paired with P<0.05 when comparing protocols.

**Fig. 3:**
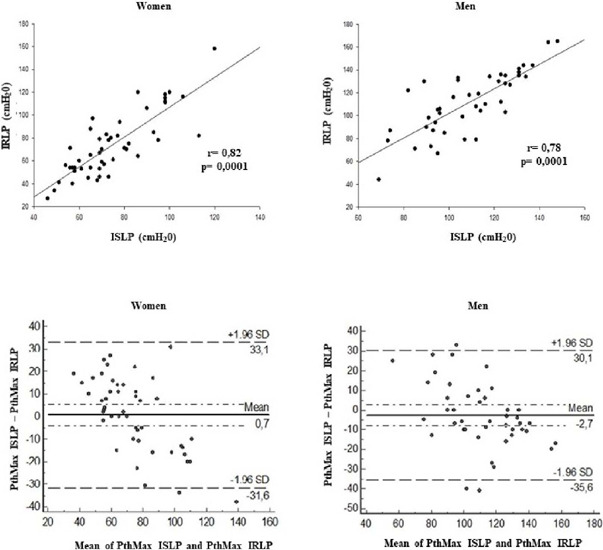
Pearson correlation and Bland-Altman agreement between incremental step loading protocol (ISLP) and incremental ramp load protocol (IRLP) for women and men. PthMax=maximum threshold pressure; SD=standard deviation.

**Fig. 4:**
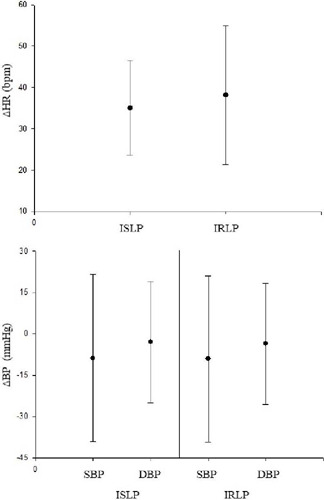
Delta of heart rate (HR), systolic blood pressure (SBP), and diastolic blood pressure (DBP) of 16 volunteers (males and females) evaluated by the incremental step loading protocol (ISLP) and the incremental ramp load protocol (IRLP) and analyzed by the paired t-test. BP=blood pressure.

One hundred and one young and eutrophic individuals were screened. Nine individuals were excluded for failing to perform S-Index. Male subjects presented a higher level of physical activity by IPAQ when compared to female subjects. None of the individuals had inspiratory muscle weakness, as shown in Table 1. Regarding the variables in men, the PthMax/S-Index (66.74 ± 7.78 and 67.86 ± 13.29 cmH₂0) and the PthMax (109.02 ± 19.68 and 111.25 ± 23.95 cmH₂0) showed no statistical difference between ISLP and IRLP, respectively. However, total time (94.04 ± 54.63 and 138.79 ± 27.52 seconds) presented a significant difference between ISLP and IRLP (*P<*0.05) (Figure 2). Regarding the variables in women, the PthMax/S-Index (66.53 ± 8.30 and 64.73 ± 17.81 cmH₂0) and PthMax (74.65 ± 17.20 and 73.92 ± 27.39 cmH₂0) showed no statistical difference between ISLP and IRLP, respectively. The total time, as in men, also presented statistical difference between ISLP and IRLP (94.53 ± 56.56 and 132.73 ± 35.55 seconds) (*P<*0.05) (Figure 2). Data from the Pearson analysis demonstrated a strong and significant correlation for the PthMax variable between ISLP and IRLP (*r* = 0.78, *P<*0.001 and *r* = 0.82, *P<*0.001, for men and women, respectively) (Figure 3). In addition, the Bland-Altman test was performed, revealing good agreement, and confirming the strong correlations of PthMax (Figure 3). Regarding the analysis of the HR and blood pressure delta, no statistical difference was observed between the protocols, which shows that even with longer total ventilatory time in the IRLP, both have similar cardiovascular stress (Figure 4).

**Table 1:** Demographics, anthropometric data, level of physical activity, and inspiratory muscle strength of the volunteers studied.

	Women	Men
n = 92	n = 49	n = 43
Age (years)	22 ± 3	22 ± 3
Body mass (kg)	57.50 ± 9.53	76.63 ± 12.82
Height (m)	1.63 ± 0.06	1.77 ± 0.08
BMI (kg/m^2^)	21.80 ± 3.60	24.50 ± 3.34
IPAQ, very active (%)	24.48	46.51
IPAQ, active (%)	40.82	39.53
IPAQ, irregularly active (%)	26.54	13.96
IPAQ, inactive (%)	8.16	0
S-Index (cmH_2_O)	111.55 ± 18.33	162.95 ± 24.15

Data are expressed as mean ± standard deviation or percentages

BMI=body mass index; IPAQ=International Physical Activity Questionnaire; S-Index=strength index

## DISCUSSION

The main findings of our study showed that the new protocol, with small increases in the load and starting from minimum values of S-Index, was able to evaluate IME through the PthMax of healthy individuals. IRLP also showed a longer total time until reaching PthMax compared to ISLP, despite both protocols presented similar cardiovascular stress. To our knowledge, this is the first study to propose a protocol in ramp conformation to evaluate IME through PthMax, making the IRLP an extremely relevant tool with greater accuracy in the stratification of the load obtained during incremental resistance tests.

In our study, IMS assessment was done through the S-Index, which is a dynamic measurement of the inspiratory muscles. In a previous study, we found that the values of S-Index are not the same as those of MIP. However, the two methods of IMS assessment had a strong and significant correlation (*r* = 0.74 and *P<*0.0001), as well as good concordance evidenced by the Bland-Altman analysis^[16]^. The rational application of S-Index, for IMS dynamic evaluation, refers to standardization of muscle contraction pattern adequate to support the dynamic endurance protocols, in contrast to protocols used in previous studies that based the resistance evaluation in a static force measurement (MIP) to support the ISLP^[6,10,12]^.

The ISLP has been a widely used tool to stratify the PthMax of individuals aiming to evaluate the impact of different IMT protocols on post-intervention IME^[6,7,9,11]^. However, the ISLP presents limitations that urges to the conception of a new strategy. The ISLP’s first limitation is the stratification in percentages per 10%, which may represent a wide range of stratification for fatigue. This is a limitation for stratification of the maximum tolerated load during the evaluation, either to analyze intervention effects or to measure PthMax for serial training. This may explain the fact that authors who apply ISLP do so only to try to evidence an improvement in IME even basing their IMT protocols on the MIP maneuver^[6,9,10]^. In contrast, in the IRLP, the increment occurs every 2% of the maximum value obtained in the previous force test. We have extremely detailed values of load stratification, not only allowing precision in the post-intervention evaluation, but also as a possible variable when prescribing the appropriate load for the IMT.

The second limitation is to evaluate individuals with low resistance through ISLP. Since it begins with loads of 50-60% of the maximum pressure value obtained in the IMS test by varying the load every 1-3 minutes^[9,12]^, it may restrict the good evaluation capacity, as the individuals do not remain in the test for a minimum time necessary for the stratification of PthMax. This ISLP’s limitation can be observed by the high standard deviation observed in the time variable of our study which demonstrates the selectivity of the test to individuals with higher levels of IME. Alternatively, in the IRLP, the increase of loads starts from minimum values, which allows its application to individuals who present all degrees of IME.

In the present study, healthy individuals required a longer total time to obtain PthMax performing IRLP with similar cardiovascular stress and safety of ISLP. However, further studies of these protocols are necessary in other populations, protocols with serial inspirations against high resistances that can determine impacts on the cardiovascular system of special populations. Scharf et al.^[22]^ demonstrated that in individuals with coronary artery disease or previously infarcted submitted to multiple inspirations against occluded airway, a left ventricular wall akinesia could occur, which impairs ejection fraction and increases SBP and DBP. Sampol et al.^[23]^ showed that high intrathoracic negative pressures increase the sympathetic activity and overload of the intrathoracic structures, which can lead to increased aortic transmural pressure and dilation, as well as posterior dissection, clinically evidenced in individuals with obstructive sleep apnea and Marfan syndrome. Taking this into account, we suggest that future studies evaluate whether IRLP could be more adequate to cardiovascular patients since it has a slow progression and a shorter total charge time allowing better adaptation to the imposed overload. Another relevant aspect of the IRLP was the report of the great majority of individuals in qualifying it as a “less unpleasant” tool. From this, it is possible to suppose that neuroadaptive factors, correlated to the small increment, allowed the individuals to be better adapted to the progression of the loads.

### Limitations

A limitation of our study was not to have inserted a tool that checked a subjective response on the adaptation to each protocol, being made by simple asking the volunteers after each test.

## CONCLUSION

In conclusion, our study suggests that IRLP is able to measure PthMax in a more thorough way, with less progression and greater accuracy in the load stratification compared to the limited ISLP and with a safe and expected cardiovascular response in healthy individuals.

## Abbreviations, Acronyms & Symbols

BMI: Body mass index
BP: Blood pressure
CHF: Chronic heart failure
DBP: Diastolic blood pressure
HR: Heart rate
IME: Inspiratory muscle endurance
IMS: Inspiratory muscle strength
IMT: Inspiratory muscle training
IPAQ: International Physical Activity Questionnaire
IRLP: Incremental ramp load protocol
ISLP: Incremental step loading protocol
MIP: Maximum inspiratory pressure
PthMax: Maximum threshold pressure
S-Index: Strength index
SBP: Systolic blood pressure
SD: Standard deviation
